# Action on the Death of Dr. Jewett

**Published:** 1911-01

**Authors:** 


### Action on the Death of Dr. Jewett.

AT a meeting of the council of the Medical Society of the State of New York held at Albany, December 3, 1910, the following memorial was adopted and ordered spread upon the records:

### IN MEMORIAM.

### Dr Charles Jewett.

In the death of Dr. Charles Jewett, not only has the Medical Society of the State of New York lost its honored president, but the medical profession of this country will miss from its ranks a tireless worker, a distinguished teacher, and an eminent surgeon.

In the fulness of years and the ripeness of experience there was conferred upon him his last public honor—the presidency of the state society. Unsought and undesired, it was conferred by his colleagues as a fitting recognition of his distinguished achievements and his sterling worth.

However distinguished the office there was something about the man that no office could compass, and no honor could magnify. For whether in the councils of the mighty, or in the homes of the humble, his services were always distinguished, and his judgments clothed with authority.

Among obstetricians he was a leader by right of worth and superiority, by force of character and quality of achievement.

He crystallized his knowledge into literature and enriched his profession by a fruitage that will live as a permanent asset.

Because of his ability to sift, correlate, clarify, and vitalize the truth, he was a teacher of power and preëminence; and a host of students throughout the world are the living witnesses of his scholarly and forceful personality.

Because of his advanced thought and judicial mind, his counsel and advice were sought by his brethren in the perplexities of human suffering, and the depth of his knowledge and the strength of his character were always an abiding inspiration.

The Medical Society of the State of New York sorrowing in the loss of its distinguished President humbly voices its appreciation of his work and his worth; and thus again renews its faith in the permanency of character, the supremacy of service, and the triumph of worth.

William Francis Campbell, M. D.,

E. B. Cragin, M. D.,

J. A. Sampson, M. D.

---

Dr. F. E. Fronczak, health commissioner, has arranged with the moving picture show places in Buffalo, to set apart Wednesday, December 28, 1910, as tuberculosis day. The managers of nearly all these theaters have offered to donate the net proceeds of that day's business to the Buffalo Association for the Relief and Control of Tuberculosis. Arrangements are under way to make it a banner day. In addition to the special slides furnished by the health department, the theaters will run an attractive program of a type, combining the educational and amusement features and thus appeal to the average audience.

The health department and the Buffalo Association for the Relief and Control of Tuberculosis are co-operating to make December 28 count in the fight against tuberculosis.

---

The danger of flies as carriers of disease germs always should be kept in mind, and physicians should never fail on proper occasions to accentuate this fact, that their patients and friends may be made to understand the fact in order to abate the nuisance. The importance of such knowledge was dwelt upon by Dr. H. D. Pease, sanitary expert of the New York City Board of Water Supply, who told his hearers recently in the Horace Mann Auditorium something about flies as carriers of disease.

He said in all his experiences he had never been able to trace an epidemic of typhoid fever directly and solely to the city's water pollution. In all such cases the germ was present from some other source and was brought there by some carrier of germs—probably flies.

"There is no use disputing the fact that flies have been one of the most dangerous elements in the cause and spread of disease, particularly in war," said the lecturer. "A recent epidemic of cholera in Berlin was traced directly to this source, while another smallpox epidemic spread to its greatest extent as a result of this carrier. It is admitted that in war typhoid has been brought to the camps by flies, which got into the provisions that were eaten later by the soldiers. In the Spanish-American War 90 per cent. of the typhoid fever cases were due to fly transmission, while 80 per cent. of the whole deaths of the war were from typhoid."

---

JUSTICE LE BOEUF, of the Supreme Court at Albany, handed down a decision recently holding that municipalities have no right to polute public water courses through sewage.

"The policy of this state," said Justice Le Boeuf, "absolutely requires that watercourses of this character shall be maintained in a pure and wholesome condition. The rights and health of the citizens of the state are dependent upon the enforcement of this policy. The individual riparian owner is entitled to the maintenance of this condition of the stream, not only as against individuals, but against a municipal corporation. No proscriptive rights on the part of an individual or on the part of a municipal corporation exist to create a public nuisance detrimental to the rights of riparian owners.

"The state in granting aid to municipal corporations to install sewer systems cannot and does not confer upon them the right to maintain a municipal nuisance of this character, except in such cases as permit condemnation upon making due compensation."

---

IN an article in *The American Magazine,* Dr. William Osler pays a high tribute to the work of Colonel Gorgas and the other medical officers of the Panama Cañal. "Of more than fifty-four thousand employes about thirteen thousand of whom are white," says Dr. Osler, "the death rate per thousand for the month of March was 8.91, a lower percentage, I believe, than in any city in the United Kingdom, and very much lower than in any city in the United States. It has been brought about in great part by researches into the life history of the parasite which produces malaria and by the effective measures taken for its destruction."

A JURY in the Supreme Court at Long Island City, according to a press despatch, returned a verdict for $8,000, December 12, 1910, against Dr. Pedro Franke and Dr. T. Grover de la Hoyde, formerly on the staff of Saint Joseph's Hospital at Far Rockaway, on a complaint that they performed an autopsy on the body of James Allen Boyd, a victim of appendicitis, without having obtained the consent of his family. Two sons and a daughter brought the suit. This would seem to be a pretty heavy verdict for such an offense.

---

JUDGE ELY of the Boston Municipal Courts has raised the fine for spitting on the sidewalk from $2 to $5 and said that unless the "disgusting and unhealthy habit" was stopped he would raise the fine still higher.

Among the first victims of the increase was Angelo Carpenilla, one of the court interpreters. He spat in the corridor outside of the room where Judge Ely was holding court. Patrolman Marks, who was there in plain clothes for the purpose of catching offenders, arrested him and took him into the courtroom. Carpenilla pleaded guilty and paid his $5 fine. It would be well if a similar method of dealing with such offenders were adopted in Buffalo.

---

DR. F. E. FRONCZAK, health commissioner of Buffalo, recently announced the appointment of Dr. James L. McGill, of No. 23 Mariner Street, to be a food and drug inspector in the health department. Herbert M. Dowling of No. 1131 Main Street was appointed to replace Dr. William B. May as milk inspector. Dr. Hugh J. McGee of No. 820 Elk Street was appointed to succeed Dr. Ludwig O. Thoma, resigned, as assistant medical school inspector. Dr. May, it will be remembered, has become attached to the State Department of Health as chief of the division of communicable diseases, as noted in the JOURNAL for December.

---

MOVING pictures as a means of curing insane patients are being tried by Superintendent Sidney D. Wilgus, of the Elgin (Ill.) State Hospital. The first pictures were shown on Christmas Day and two exhibitions a week will be given thereafter.

"Moving pictures will help us materially in curing patients," said Dr. Wilgus, in a recent conversation. "They will take the minds of patients from their misfortunes and like any other harmless diversion will stimulate their weakened brains." Dr. Wilgus, formerly of this city, is a graduate of the Medical Department of the University of Buffalo.

A crusade against selling rotten eggs for food was recently begun by Dr. F. E. Fronczak, health commissioner of Buffalo. As a result of a vigorous inspection made of the cold-storage warehouses, five tons of eggs were seized recently by the department of health and their use for anything but tanning forbidden. The eggs were decomposed, the department claimed, and were unfit for baking purposes, for which they were intended.

John Lord O'Brian, United States attorney for this district, is joining in the crusade. He secured a conviction of the Henry Sloan Company not long ago for shipping a consignment of rotten eggs intended for use in bakeshops. Judge Holt, who was holding the term of federal court here at the time, imposed the limit sentence for a single violation of the pure food law, $200.

Let the good work go forward until the last bad egg is smashed and the vendors punished to the maximum permitted by law.

---

A despatch sent from Saint Petersburg about a month ago stated that the whole of Manchuria was officially declared to be infected with the bubonic plague, but not with cholera, as was erroneously reported recently. Stringent measures have been taken on the Siberian frontier to prevent the entrance of the epidemic into Primorskaya. Supplies of serum have been sent to Vladivostok.

---

The superintendent of schools, Mr. Henry P. Emerson, announces that he is in favor of open-air schools for children who incline toward tuberculosis, and already one such school has been started. It is a wise plan, having been successful in Germany, where it originated, as well as in many cities of this country, where already it has been tested. In New York the roofs of two-story public baths have been utilised at small expense for construction, the one near Hudson Street park costing but $6,000 to prepare for fifty pupils.

---

Magistrate House, in the Yorkville Court (New York), recently fined fourteen automobilists $5 each because their machines were causing the atmosphere to be decorated with clouds of odorous discharge. He added that if the imposing of fines did not stop the nuisance he would hand down prison sentences.

First, however, the Magistrate will raise the fine to $10 for each offense, then, if there is no alleviation, he will make good his prison threat.

